# Oncological Aspects of Lysosomal Storage Diseases

**DOI:** 10.3390/cells13191664

**Published:** 2024-10-08

**Authors:** Agnieszka Ługowska

**Affiliations:** Department of Genetics, Institute of Psychiatry and Neurology, Al. Sobieskiego 9, 02-957 Warsaw, Poland; alugipin@yahoo.com

**Keywords:** lysosomal storage diseases, cancer, lysosomal hydrolases

## Abstract

Lysosomal storage diseases (LSDs) are caused by the deficient activity of a lysosomal hydrolase or the lack of a functional membrane protein, transporter, activator, or other protein. Lysosomal enzymes break down macromolecular compounds, which contribute to metabolic homeostasis. Stored, undegraded materials have multiple effects on cells that lead to the activation of autophagy and apoptosis, including the toxic effects of lyso-lipids, the disruption of intracellular Ca^2+^ ion homeostasis, the secondary storage of macromolecular compounds, the activation of signal transduction, apoptosis, inflammatory processes, deficiencies of intermediate compounds, and many other pathways. Clinical observations have shown that carriers of potentially pathogenic variants in LSD-associated genes and patients affected with some LSDs are at a higher risk of cancer, although the results of studies on the frequency of oncological diseases in LSD patients are controversial. Cancer is found in individuals affected with Gaucher disease, Fabry disease, Niemann-Pick type A and B diseases, alfa-mannosidosis, and sialidosis. Increased cancer prevalence has also been reported in carriers of a potentially pathogenic variant of an LSD gene, namely *CLN3*, *SGSH*, *GUSB*, *NEU1*, and, to a lesser extent, in other genes. In this review, LSDs in which oncological events can be observed are described.

## 1. Introduction

Rare hereditary metabolic diseases include a group of lysosomal storage disorders (LSDs). They are caused by the deficient activity of a lysosomal hydrolase or a lack of a functional lysosomal membrane protein, transporter, activator, or other protein. The role of lysosomal enzymes is the degradation of macromolecular compounds, which contributes to metabolic homeostasis. This catabolic activity of lysosomes, which are small structures suspended in the cytoplasm, allows them to be considered a cellular recycling center.

Lysosomes are the main compartment associated with cellular digestion. They are organelles surrounded by a single protein-lipid bilayer membrane and are found in all cells of mammalian organisms except erythrocytes. Unlike other cellular organelles, lysosomes cannot be recognized on the basis of the usual morphological criteria—size, shape, and internal structure—as they show great polymorphism. Using electron microscopy, lysosomes are defined as dense bodies of a spherical, oval, or tubular shape containing an osmiophilic matrix. The size of lysosomes ranges from about 1 to several μm. Both the size and number of lysosomes can increase in any cell type when undegraded macromolecular compounds are accumulated.

Lysosomes in animal cells were first identified by de Duve and colleagues in 1955 [1]. These organelles were named lysosomes, meaning digesting or lytic bodies. Lysosomes have been found in the cells of protozoa, insects, amphibians, and mammals, as well as in plant cells [2,3].

More than 60 hydrolytic enzymes that digest proteins, nucleic acids, polysaccharides, lipids, phospholipids, and sulfates have been detected in lysosomes [4]. Macromolecular compounds, which are substrates of the action of lysosomal hydrolases, are delivered via endocytosis, phagocytosis, and autophagy. The small-molecule compounds released in the degradation process are transported to the cytoplasm and reincorporated into metabolism. The lysosomal membrane contains many transporters that enable the movement of amino acids, saccharides, and probably nucleotides into the cytoplasm. The lysosomal membrane separates lysosomal hydrolytic enzymes and prevents uncontrolled lysis of cytoplasmic components [5]. The low pH inside the lysosome is ensured by the presence of a proton pump belonging to H^+^-ATPases in the lysosomal membrane. The presence of the highly glycosylated proteins LAMP1, LAMP2, LIMP1, and LIMP2, anchored in the membrane, protects it from the hydrolytic action of enzymes [6].

The precursors of most lysosomal enzymes, after synthesis on the rough endoplasmic reticulum (RER) and modification in the Golgi apparatus, are equipped with mannose-6-phosphate (Man-6-P) markers that can bind to Man-6-P receptors (MPRs) located in the trans Golgi network (TGN) [7,8,9]. There are two types of MPRs: cation-independent (CI-MPR) and cation-dependent (CD-MPR). The lysosomal enzyme precursors are then packaged into clathrin-coated vesicles (CCVs) and transported to late endosomes (LEs), either directly or indirectly through early endosomes (EEs). The process of transferring the enzyme from the LE to the lysosome is not yet fully elucidated. It is probable that the LE matures and becomes a lysosome, or the LE and lysosome undergo fusion and form a transient hybrid organelle. MPR receptors recycle from the LE into the TGN, while lysosomes are devoid of these. A small fraction of lysosomal enzyme precursors enter the secretory pathway, are released outside the cell via constitutive secretion, and are re-captured by MPRs into clathrin-coated pits (CCPs) in the cell membrane. Enzyme precursors can also reach lysosomes via endocytosis. Lysosomal enzymes enter autophagic vacuoles (AVs) and phagocytic vacuoles (PVs) via the fusion of lysosomes or LEs with these vacuoles. In addition to the MPR-mediated transport of lysosomal enzymes, there are poorly understood alternative mechanisms independent of Man-6-P receptors, e.g., β-glucocerebrosidase enters lysosomes via the LIMP-2 protein [10].

As a result of pathogenic variants, a particular lysosomal enzyme may be absent or drastically reduced in activity. This entails the accumulation of large-molecule, undegraded metabolites inside lysosomes, followed by tissue and organ dysfunction. To put it briefly, as a consequence of the above events, lysosomal disease develops, resulting from the defective activity of an enzyme localized in the lysosomal system (in its interior or membrane). The lysosomal disease can also be an effect of impaired function of cofactors, activator proteins, transporters, proteins that build ion channels and the lysosomal membrane, and proteins involved in the posttranslational processing and communication of lysosomal enzymes with other organelles, e.g., the nucleus and mitochondria [11,12]. The term “lysosomal disease” was first used by Hers to refer to type II glycogenosis (Pompe disease) [13]. Like other genetic defects, LSDs probably affect the metabolism of all cell types in the body, in which the damaged enzyme or other protein is controlled by the same gene. In his concept of inborn lysosomal diseases; however, Hers emphasizes that disease symptoms may proceed with different intensities depending on the tissue, which is related to the rate of metabolite storage, the secretory capacity of the cell, and its lifespan [14]. Other yet undefined genetic and/or environmental factors may also influence the clinical picture of the disease.

Lysosomes are involved in the processes of exo- and endocytosis and autophagy. They participate in the maturation of autophagic vacuoles and continue the degradation of proteins and organelles initiated by autophagy. In postmitotic cells, such as neurons whose lysosomal functions were damaged or in lipofuscin-storing aging cells, the accumulation of inefficient, “weak” mitochondria has been demonstrated. In various lysosomal diseases, the disorder of the lysosomal–mitochondrial axis leads to destruction in homeostasis maintenance and finally to cell death (in great simplification). According to some theories, deficiencies in the function of enzymes and other lysosomal proteins in lysosomal diseases inhibit the maturation of autophagic vacuoles, thus causing autophagic stress. This leads to the accumulation of fragmented, partially inefficient mitochondria with a defective system of buffering of Ca^2+^ ions, whose local increase in concentration can activate calpain and, subsequently, the caspase cascade, triggering the process of apoptosis. The cause of apoptosis in the case of cells with lysosomal disruption would not only be the accumulation or overloading of lysosomes with stored, undegraded substrates but the accumulation of damaged mitochondria in the cytoplasm of the cell due to the disruption of the autophagy process [15]. Restricted degradation of damaged mitochondria through different mechanisms may contribute to LSD pathogenesis. This degradation is performed either in a selective way—mitophagy, or in a non-specific way—macroautophy [16,17].

Thus, lysosomes are not just a cellular “stomach”. It seems that lysosomes have more important functions in cellular metabolism than previously thought. In particular, participation in the recycling and degradation of macromolecular compounds is crucial for maintaining cellular homeostasis. Walkley explains this using the example of Niemann-Pick type C disease, in which a lack of functional NPC1 or NPC2 proteins leads to impaired free cholesterol recycling in the endosome system. In healthy cells, the by-products of cholesterol metabolism are oxysterols, which play a role in cholesterol synthesis. The lack of oxysterols in NPC cells impairs the homeostasis of cholesterol synthesis and distribution in NPC neurons. The pathogenetic factor in Niemann-Pick type C disease, and probably other lysosomal diseases, is not the overloading of lysosomes with undegraded substrate but the lack of some by-products, which are essential for the synthesis of new compounds and regulation of cellular metabolism, and which are normally formed from macromolecular compounds in healthy cells. Understanding the pathomechanism of lysosomal diseases in this way allowed Walkley to propose a new approach to treating these diseases. Supplementing missing by-products or intermediates of metabolism or administering drugs that can maintain normal neuronal metabolism is the main idea behind BRT (by-product replacement therapy) [18]. Other treatment strategies include enzyme replacement therapy (ERT), pharmacological chaperone therapy (PCT), substrate reduction therapy (SRT), hematopoietic stem cell transplantation (HSCT), gene therapy, genome editing, proteostasis regulators, small-molecule-assisted substrate transportation (SMAST), anti-inflammatory agents. For more information on lysosomal biogenesis and function, as well as their connection to nutrient sensing, please refer to the following review articles: (1) [19], (2) [20], (3) [21].

Apart from catabolic functions, lysosomes also have anabolic functions. It has been shown that in damaged cells, lysosomes fuse with the cytoplasmic membrane and replenish its deficiencies through the process of Ca^2+^ ion-dependent exocytosis. Thus, lysosomes serve as a spare reservoir of membrane lipids, when rapid synthesis of adequate lipids is impossible [22,23].

Stored, undegraded materials display diverse influences on cells. Previous scientific results indicate that autophagy and apoptosis processes are activated in individual lysosomal diseases. The toxic effects of lyso-lipid compounds, disruption of intracellular Ca^2+^ ion homeostasis, secondary storage of macromolecular compounds, activation of signal transduction and inflammatory processes, deficiency of intermediate compounds, and many others have been demonstrated (for a review, please see [24]).

Presently, over 80 inherited diseases connected with the lysosomal system are distinguished (summarized in Supplementary Table S1). When considered as a group, the incidence of lysosomal diseases (LSDs) oscillates between 1 in 5000 to 1 in 7700 live births, whereas the incidence of individual LSDs may range from 1 in 57,000 for Gaucher disease to as low as 1 in 4.2 million for sialidosis [25,26]. Thus, all LSDs fulfill the criteria for rare diseases, with incidence estimated in Europe to be less than 1 in 2000 and in the USA less than 1 in 1650 live births [27,28].

The clinical picture of lysosomal diseases (LSDs) is very heterogeneous and without characteristic or specific signs or symptoms. This makes the diagnostic process difficult and demands the cooperation of various specialists. The majority of LSD display, however, some basic clinical manifestations, which include the following:

- chronic, progressive nature of the disease;
- in the neonatal period, often symptoms not very pronounced;
- sometimes hydrops fetalis;
- dysmorphic facial features;
- skeletal changes (especially dysostosis multiplex, Erlenmayer’s beaker type deformity of long bones);
- skin lesions (e.g., angiokeratoma);
- muscular hypotonia;
- delayed motor and then mental development;
- progressive organomegaly (liver, spleen, heart);
- features of leukodystrophy (ataxia, hyperactivity, spasticity, paresis);
- cortical and subcortical lesions;
- pyramidal and extrapyramidal symptoms;
- polyneuropathy;
- cerebellar ataxia;
- epilepsy of unclear etiology;
- hearing loss, vision loss;
- cherry red spot on the fundus, corneal opacity, lens subluxation [29].

Interestingly, clinical observations have shown that patients affected with some LSDs and carriers of potentially pathogenic variants in LSD genes are at higher risk of cancer. In this article, LSDs, in which oncological events have been reported, are described to provide a clearer picture of this putative LSD-cancer association (Table 1). While it is known that the hyperexpression and/or hypersecretion of some lysosomal enzymes can promote cancer progression (reviewed by [30,31,32] and summarized in Supplementary Material File S1), little is known about the putative links between lysosomal storage conditions and cancer.

The importance of lysosomes in cancer cells cannot be ignored, and there are outstanding reviews summarizing the knowledge on this subject. A few essential pieces of information are recapitulated in Supplementary Material File S1.

## 2. Gaucher Disease

The lack of functionally active beta-glucocerebrosidase, which is one of the lysosomal hydrolases, leads to the intra-lysosomal storage of glucocerebroside (GlcCer), glucosylsphingosine (GlcSph) and sphingosine-1-phosphate (S-1-P) in the cells of monocyte/macrophage lineage. These lipid-laden macrophages are described as ‘Gaucher-cells’, and they can infiltrate some organs, e.g., the liver, spleen, and bone marrow. The storage of undegraded lipid compounds causes pathogenic events and the launch of replacement metabolic pathways in Gaucher disease (GD). Pathogenic variants in the *GBA1* gene result in the deficient activity of beta-glucocerebrosidase (GC-ase, glucosylceramidase, lysosomal beta-glucosidase). Very rarely, GD can be the effect of functionally inactive protein activator of glucocerebrosidase, saposin C (SAP-C), due to mutations in the *PSAP* gene. In both cases, GD is inherited in an autosomal recessive manner.

Although the phenotype displays a wide spectrum, three main clinical types of GD are distinguished:

- Type 1—non-neuronopathic, bone involvement (bone pains, osteopenia, pathological fractures, deformity of long bones known as ‘Erlenmayer flask’ bones).
- Type 2—neuronopathic, severe lung involvement.
- Type 3—neuronopathic, slowly progressive, supranuclear horizontal gaze palsy.

All types are characterized by hepatosplenomegaly and pancytopenia, as well as the presence of activated macrophages and Gaucher cells in the bone marrow.

Although the increased risk of malignancy in patients with Gaucher disease has been reported earlier, Rosenbloom et al. performed a study on a large cohort of 2742 patients from the International Gaucher Registry [34]. They identified 10 patients with multiple myeloma with a significantly higher risk than in the general population (estimated relative risk (RR) of 5.9). Obtained results indicated that, in general, GD patients are not at a higher risk for various types of cancer (RR 0.79) except for multiple myeloma [34]. In the next study, from 2022, Rosenbloom found that the risk for hematological malignancies was more than 4 times higher in a group of 2123 GD patients in comparison with the general population: non-Hodgkin lymphoma was approximately three times higher, multiple myeloma was approximately nine times higher. Monoclonal gammopathy of unknown significance (MGUS) was observed more frequently among younger GD patients [35]. Additionally, in this group of patients, Rosenbloom et al. found higher risks for solid malignancies of the liver (2.9 times), kidney (2.8 times), melanoma (2.5 times), and breast (1.4 times), while colorectal, prostate, and lung cancer risks were lower than expected [35].

Different results were found by Landgren et al. in a group of 1525 patients with Gaucher disease, in whom the relative risk for non-Hodgkin lymphoma (RR, 2.54), malignant melanoma (RR, 3.07), and pancreatic cancer (RR, 2.37) was elevated [36]. Unlike in the group studied by Rosenbloom et al., in this group of patients, there was no significant association between Gaucher disease and cancer in general or multiple myeloma.

On the basis of the reviewed literature, Choy and Campbell emphasized that multiple myeloma has been most frequently reported, and the increased risk of this malignancy was particularly noted in homozygotes for the p.N370S mutation in the *GBA1* gene (RR, 25) [37,38]. Other kinds of cancers reported in GD patients included chronic lymphocytic leukemia, chronic myeloid leukemia, acute leukemia, large B-cell lymphoma, T-cell lymphoma, Hodgkin’s disease, glioblastoma multiforme, lung cancer, dysgerminoma, hepatocellular carcinoma, and bone cancer (for details, please see Table 1 in [37]). Hughes cited the problem of patients with multiple carcinomas. Additionally, she concluded that the risk of malignancies seems to be highest in patients who had undergone splenectomy [39].

Bozdag et al. described a GD patient who developed acute lymphoblastic leukemia (ALL) while being treated with enzyme replacement therapy (ERT) [40]. Lo et al. suggested that ERT targets only macrophages whereas pathologic processes continue in other types of cells, which may be the source for carcinogenesis [40,41]. Mishra et al. diagnosed the co-existence of Gaucher disease and acute lymphoblastic leukemia in a 1.5-year-old female child who was presented to a hospital because of a gradually increasing lump in the left upper abdomen. This patient was anemic with massive splenomegaly. In the bone marrow aspirate, Gaucher cells, as well as atypical small round cells, were visible. Immunohistochemical analysis allowed the recognition of acute precursor B-cell lymphoblastic leukemia [42].

Baris et al. pointed out that in numerous studies, the relative risk of multiple myeloma (RR, 5.9) and non-myeloma hematological malignancies is increased in GD patients, but the relative risk of cancer in general did not differ from the general population (RR, 0.79). The exceptions are hepatocellular carcinoma, renal cell carcinoma, and melanoma [43].

In a cohort of 131 GD patients from the German and Dutch populations, de Fost et al. observed an increased risk of cancer (2.5) and an increased risk of hematologic cancer (12.7) in affected individuals in comparison to the general Dutch population [44].

According to Monge, an extended diagnostic procedure should be performed in the case of abnormal bone marrow signal because pseudo-Gaucher cells (histiocytes with monoclonal immunoglobulin crystals) can be found in multiple hematologic malignancies, hemoglobinopathies, infections, and multiple storage disorders. Especially, differentiating Gaucher disease from multiple myeloma is important. Additionally, in GD patients, there is a higher risk of polyclonal and monoclonal gammopathies and multiple myeloma, as was already mentioned. Thus, the co-existence of these two conditions cannot be excluded. ERT may result in an improvement in polyclonal gammopathies, but its influence on the effect of the progression of monoclonal gammopathy of undetermined significance to active multiple myeloma remains uncertain [45,46].

Ridova et al. described a rare case of early-onset colorectal cancer in a young woman diagnosed with GD type 1 in early adulthood, shortly after GD diagnosis was made and ERT was started. In this patient, radical surgical resection of the colon and adjuvant chemotherapy were applied. Due to cytopenia, anticancer treatment in patients with GD demands a peculiar approach [47].

Choy and Campbell summarized that although some kinds of cancers, especially myeloma, are more frequent in GD patients than in the general population, the frequencies of the other kinds of cancers remain on the average levels seen in the population (particularly in young patients) [37].

Pastores and Hughes point out that Gaucher-like or pseudo-Gaucher cells can be seen in acute lymphoblastic leukemia, multiple myeloma, myelodysplasia, and Hodgkin’s disease [48].

### Theoretical Relationship between GD and Cancers

Dubot et al. emphasize that the higher frequency of diverse kinds of cancer in GD patients did not differ between patients receiving treatment (like ERT, the substrate reduction therapy—SRT) or not. Some hypotheses elucidating the causes of co-occurrence of Gaucher disease and cancer have been proposed. In GD individuals, both chronic cell and tissue inflammation, as well as immune system dysregulation, have been noted. In plasma samples from affected patients, increased levels of pro- and anti-inflammatory cytokines, including IL-1β, TNFα, IL-8, IL-10, and IL-6, which regulate B-cell proliferation were found, which could enable a potential transformation to a neoplasm [37,49]. Interestingly, Gaucher cells containing stored undigested material, i.e., GlcCer and GlcSph, produce anti-inflammatory cytokines and chemokines, leading to an immune cell infiltration (with macrophages, dendritic cells, neutrophils, and lymphocytes) in organs of GD mouse models [49]. According to Dubot et al., GlcCer and GlcSph seem to play a crucial role in GD predisposition to cancer diseases because of their involvement in processes of inflammation, immune activation, and signaling [49].

It was also proposed that a higher frequency of cancer in GD patients may be the effect of activated autophagy and cell death processes, in which GCase was shown to be a positive mediator. Dasari et al. performed an experiment in which lung cancer cells were treated with resveratrol in order to induce autophagic cell death. In these conditions, they observed a 3-fold increase in GCase protein quantity and elevated activity of this enzyme. Silencing the expression of *GBA1* by transfection with siGBA1 reduced the sensitivity of lung cancer cells to induced autophagic-dependent cell death, thereby promoting cell survival. As already mentioned, GCase catalyzes the reaction of GlcCer degradation to Cer and glucose. Ceramide is then metabolized to sphingosine and sphingosine-1-phosphate (S-1-P) [50]. It seems that changes in the lipid composition and proportion may play a role in the vulnerability of GD patients to cancer. Ceramide, which is released by GCase from GlcCer, is a tumor suppressor. It can be speculated that low levels of this compound would promote survival of the tumor cells [51]. Moreover, disturbed autophagy could result in a constant inflammasome activation in human Gaucher macrophages, leading to systemic inflammation [49,52].

Interestingly, protein misfolding was linked with higher melanoma incidence in patients with Parkinson’s disease (PD). Similarly, protein misfolding could explain the predisposition of GD patients and carriers of mutations in the *GBA1* gene to melanoma and PD. It has been suggested that aggregates of alfa-synuclein, which were found in the skin of patients affected with melanoma, could halt the autophagy [49,53]. On the other hand, mutated parkin could be responsible for disturbed proteasome-dependent degradation of misfolded proteins or enhancement of cell proliferation due to loss of tumor suppressor function [49,54]. Choy and Campbell speculated that misfolded GCase molecules may be retained in the endoplasmic reticulum (ER), leading to ER stress and proteasomal overload with mutated enzyme aggregates [37].

Barth et al. indicate that some authors suggest SRT to account for increased frequency of some kind of cancer in GD individuals. A limited amount of glycosphingolipids may stop potential iNKT cell-mediated antitumor activity (iNKT = invariant natural killer T cells) and anti-inflammatory activity [55]. On the contrary, the results of one study revealed that the prevalence of cancer in GD patients treated with ERT remained unchanged or has even dropped [56].

Choy and Campbell point out that dysregulated immune system may play a role in the susceptibility of GD patients to some cancers. Namely, the CD1d complex, which serves to recognize glycolipids, was elevated in NKT cells [37].

**Figure 1:**
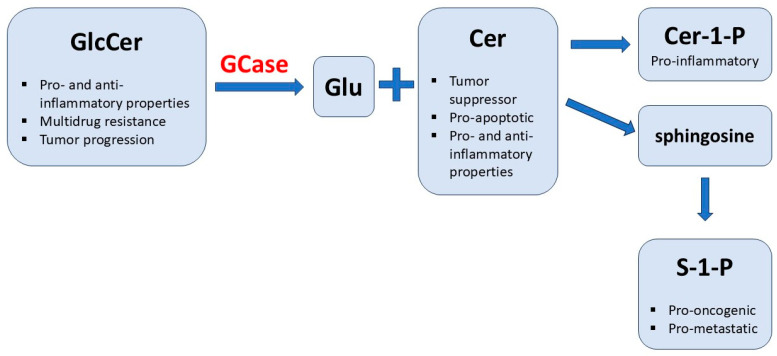
Bioactive properties of sphingolipids (GlcCer and its derivatives). GlcCer—glucocerebroside (glucosylceramide); GCase—glucocerebrosidase (lysosomal beta-glucosidase deficient in Gaucher disease); Glu—glucose; Cer—ceramide; Cer-1-P—ceramide-1-phosphate; S-1-P—sphingosine-1-phosphate.

Against the hypothesis linking the increased amounts of ceramide and susceptibility to cancer in GD patients on ERT or SRT is the fact that ceramide displays not only pro-apoptotic properties and is a mediator within pro-inflammatory pathways but also is involved in many anti-cancer strategies and exhibits anti-inflammatory features. Actually, it is the ceramide-1-phosphate, which can be a potent pro-inflammatory mediator, and S-1-P, which is a pro-oncogenic and pro-metastatic compound. It can be speculated that ERT produces excessive amounts of ceramide-1-phosphate, while SRT suppresses the synthesis of free ceramide; please see Figure 1. Stored GlcCer or other complex glycosphingolipids influence the expression of P-glycoprotein and resulting multidrug-resistance. This process does not probably stimulate carcinogenesis but may enhance the progression and severity of cancers once they occur [55]. Barth et al. propose that malignant diseases present in GD patients on ERT or SRT are likely coincidental, and the most probable cause of the occurrence of cancers in GD is the augmentation of cancer progression, not carcinogenesis, due to the pathological accumulation of GlcCer and other glycosphingolipids, Figure 1 [55].

## 3. Alfa-Mannosidosis

Alfa-mannosidosis is caused by deficient activity of lysosomal acid alfa-mannosidase due to pathogenic variants in the *MAN2B1* gene. Additionally, elevated urinary excretion of mannose-rich oligosaccharides can be demonstrated [57,58]. In light microscopy, vacuolated lymphocytes from peripheral blood are present in 90% of affected individuals [59].

Clinical signs of alfa-mannosidosis include the following:

- facial features (e.g., coarse facial features, macrocephaly, prominent forehead, highly arched brows, depressed nasal bridge, widely spaced teeth, macroglossia, prognathism);
- skeletal abnormalities;
- hearing loss;
- frequent infections;
- developmental delay;
- intellectual disability;
- ataxia [59].

Three clinical types of alfa-mannosidosis are distinguished:

- Type 1 is a benign form, with onset after the age of 10 years and very slow disease progression; skeletal abnormalities are absent.
- Type 2 is a moderate, slowly progressing form, with onset before the age of 10 years; skeletal abnormalities are present, and ataxia can be revealed by age 20 to 30 years.
- Type 3 is the severe form, with onset in early infancy, skeletal abnormalities are present, and early death may occur due to primary central nervous system involvement or myopathy [http://www.omim.org/entry/248500 (accessed on 1 August 2024)].

The affected individuals suffer from frequent bacterial, mycobacterial, and viral infections, indicating severe immunodeficiency. A possible explanation is, that accumulated mannose-rich oligosaccharides inhibit the association between the receptor IL-2Rbeta and the CD3/TCR complex leading to the restriction of IL-2-dependent signaling [60].

Structures similar to oligosaccharides reach in Man2–9GlcNAc found in alfa-mannosidosis patients were observed on C6 glioblastoma and many other malignant cells [60,61]. These N-glycans can be responsible for (1) the adhesion of cancer cells to T cells or stimulated lymphocytes expressing IL-2Rbeta; (2) an interference with the IL-2-dependent activation processes; and (3) a trapping of circulating IL-2.

It can be speculated that cancer cells would inhibit the CD8+ T cells’ differentiation into killer cells and the antigen-specific response of B cells. This could be a cause of the deficient immune response to cancer cells, a deficiency that can be restored using IL-2 therapies [60]. However, long-term treatment with oligomannoside ligands will disorganize the oligomannoside-dependent cell adhesion mechanisms and block the immune response [60].

Hennermann et al. performed a multicenter study based on a questionnaire revealing the cause of death and age at death of patients with alfa-mannosidosis. Twenty percent of reported patients died because of cancer disease, particularly—colon carcinoma, breast cancer, and leukemia [62].

**Figure 2:**
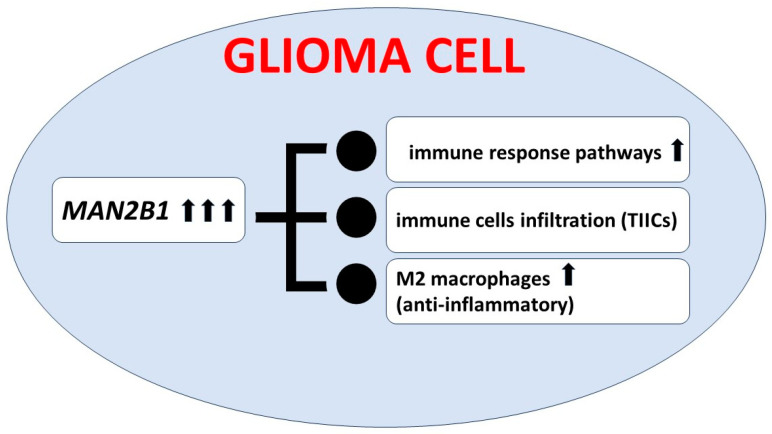
Consequences of elevated *MAN2B1* gene expression in glioma cells. TIICs—tumor-infiltrating immune cells.

Lin et al. analyzed *MAN2B1* gene expression in patients with glioma. Their results based on the RNA-seq data indicated that *MAN2B1* expression was elevated in glioma and was correlated with malignant clinical and molecular features. In patients with highly expressed *MAN2B1,* the prognosis was poor. Additionally, frequencies of somatic mutations present in gliomas with elevated and inhibited *MAN2B1* expression differed significantly. More accurate functional analyses revealed the association of high *MAN2B1* expression with markers of M2 macrophages and tumor-associated macrophages. In conclusion, the level of *MAN2B1* expression could be a prognostic biomarker in glioma and associated with immune infiltration; please see Figure 2 [63]. Observation made by Lin et al. is not directly connected to LSDs and oncological aspects, but it seems to be a very interesting and potential diagnostic biomarker.

## 4. Fabry Disease

Fabry disease is one of the lysosomal storage diseases inherited in an X-linked recessive manner. It is caused by mutations in the *GLA* gene, leading to deficient activity of alfa-galactosidase A. As a result, globotriaosylceramide (Gb3) and its derivatives, e.g., globotriaosylsphingosine (Lyso-Gb3), are stored in the lysosomes. Noteworthy, Gb3 and lyso-Gb3 are glycosphingolipids, which were detected in elevated amounts in various types of cancer. Gb3 was identified in patients with breast, colon, pancreatic, gastric, ovarian, and testicular cancers and lymphoma [64]. Interestingly, other glycosphingolipids stored in Fabry disease, lyso-Gb3 and S-1-P, promote cell proliferation [64].

Clinically, there is a spectrum of Fabry disease forms, but signs and symptoms characteristic of the most frequent classic form include attacks of bone pains, angiokeratoma, telangiectasia, sweating abnormalities, opacities of the cornea, and lenticulae. Proteinuria is a sign of disturbed renal function. Males suffering from this type of Fabry disease develop cardiac disease (mainly left ventricular hypertrophy) and stroke. The first clinical symptoms of the classic form of Fabry disease can be observed between 4 and 8 yr. of age. In late-onset forms, the beginning of the disease is in patients over 25 yr. of age, and it manifests with cardiac and renal symptoms. In symptomatic female carriers, usually milder symptoms and a later age of onset than in males are seen [65].

Bird et al. examined a group of 98 adult male patients with Fabry disease and 163 female carriers. Among them, 25 individuals were diagnosed with cancer:

- 7 cases of breast cancer in females;
- renal cell carcinoma in 2 males;
- 4 cases with melanoma; incidence rate ratio of 3.1;
- 5 cases with urological malignancies (1 with bladder cancer, 1 with ureteric cancer; incidence rate ratio of 2.7 and 3 cases with renal cancer; incidence rate ratio of 4.3).

Benign lesions were identified in 24 individuals:

- 5 cases with growths in neurological tissues (including two benign neoplasms of the meninges; the incidence rate ratio was 12);
- 5 cases with colon polyps;
- 4 cases with benign breast lesions;
- 3 cases with atypical moles;
- 2 cases with benign renal lesions;
- 2 cases with cervical intraepithelial neoplasm.

Bird et al. concluded that patients with Fabry disease, most probably, are not at highly increased risk of cancer development, although the incidence of melanoma, urological cancers, and benign meningioma is increased in them. This predisposition may be the effect of lyso-lipids action, disease-related inflammation, and vascular abnormalities [64].

**Figure 3:**
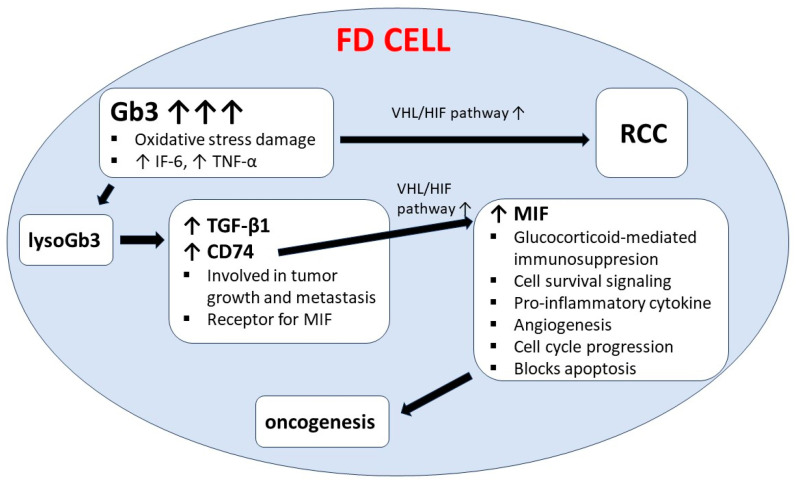
Possible correlation between renal cell carcinoma and sphingolipids stored in Fabry disease. Gb3—globotriaosylceramide; IF-6—interleukin 6; TNF-α—tumor necrosis factor-α; VHL/HIF pathway—von Hippel-Lindau/hypoxia-inducible factor 1α; RCC—renal cell carcinoma; lysoGb3—globotriaosylsphingosine; TGF-β1—transforming growth factor-β1; MIF—macrophage migration inhibitory factor; FD—Fabry disease.

Renal cell carcinoma (RCC) was also described in male Fabry disease patients by other authors [66,67]. Interestingly, Pagni et al. reported RCC in a female carrier without ERT. They hypothesized that glycosphingolipids accumulated in Fabry disease can promote cell proliferation, resulting in tumor growth. Additionally, Gb3 is involved in oxidative stress damage, which is associated with RCC development. Pagni et al. suggested a correlation between Gb3 and lyso-Gb3 accumulation, oxidative stress, and oncogenesis (through Von Hippel-Lindau/hypoxia-inducible factor 1α—VHL/HIF pathway); please see Figure 3 [68].

## 5. Niemann-Pick Disease Type A/B (Acid Sphingomyelinase Deficiency, ASMD)

Pathologic variants in the *SMPD1* gene are the cause of the deficient activity of a lysosomal hydrolase—acid sphingomyelinase (ASM), which results in Niemann-Pick disease type A or B (NPDA or NPDB). ASM is responsible for the degradation of sphingomyelin into ceramide and phosphocholine. Deficient ASM activity leads to the storage of sphingomyelin, free cholesterol, and some other lipids (bis(monoacylglycero)phosphate (BMP) and lysosphingomyelin = sphingosine phosphocholine, glucocerebroside, lactosylceramide, and gangliosides, e.g., ganglioside GM3) in the lysosomes of macrophages, hepatocytes and other cells of reticuloendothelial system. These cells, containing stored material, form the so-called foam cells [69].

NPD is inherited in an autosomal recessive manner. Clinically, NPDA and NPDB are different. NPDA is a severe neurodegenerative disease with hepatosplenomegaly. The onset of clinical signs and symptoms is in the infantile period (until about 6 months of age), and affected children usually do not survive longer than 3 yrs. Hypotonia, failure to thrive, and a ‘cherry red spot’ on the macula are characteristics. In patients with NPDB, the neurologic system involvement is not obvious. Hepatosplenomegaly and liver disease, lung involvement, ‘cherry red spot’ on the macula, and a reddish-brown halo surrounding the macula can be observed. The NPDB progresses slowly and can be accompanied by lipids disequilibrium in serum (increased levels of triglycerides and LDL-cholesterol, decreased level of HDL-cholesterol). First symptom can be hepatosplenomegaly, diagnosed from early childhood until the sixth decade of life. Due to the fact, that there exists a spectrum of phenotypes, the NPD type A/B is also distinguished. The clinical picture includes signs and symptoms of NPDB and is accompanied by neurological involvement (hypotonia, hyporeflexia) [69,70].

Mauhin et al. found cancer in 5 out of 31 patients with ASMD: in 1 female breast cancer and in 4 males two lung cancers (metastatic neuroendocrine small-cell lung carcinoma and metastatic well-differentiated squamous cell carcinoma), one papillary thyroid cancer and one bladder cancer (non-metastatic high-grade urothelial carcinoma of the bladder), giving a prevalence of 16.1%. Mauhin et al. have observed that oncological diseases were significantly associated with splenectomy [71]. Other authors also reported cases of cancer in ASMD patients: a marginal zone lymphoma, liver cancers, multiple myeloma, chondrosarcoma, and unknown type of cancer [72,73,74], cited after [71].

As mentioned above, sphingomyelinase catalyzes the breakdown of sphingomyelin into ceramide and sphingosine, which can, in turn, be metabolized into Cer-1-P and S-1-P, displaying pro-proliferative features (please, see the ‘Gaucher disease’ Section). This could explain the effect of low sphingomyelinase activity on carcinogenesis in ASMD patients [71].

## 6. Sialidosis

Gene *NEU1* codes for a lysosomal enzyme sialidase (also named alfa-N-acetylneuraminidase or neuraminidase), which removes terminal sialic acid (alfa-N-acetylneuraminic acid) residues from molecules of glycoproteins and glycolipids. Together with beta-galactosidase and cathepsin A (protective protein), it forms a heteromeric complex in which it displays activity in lysosomes. Sialyloligosaccharides are stored in cells and tissues of affected individuals and are excreted in urine.

Pathogenic variants in the *NEU1* gene result in lysosomal autosomal recessive disorder—sialidosis. Two main types of this disease are distinguished: sialidosis type 1 and sialidosis type 2. Type 1 is rather late onset, with no dysmorphic signs, but with symptoms from the nervous system, e.g., cherry red spots in the macula, myoclonic seizures, intentional tremors, cerebellar ataxia, and hyperreflexia. Type 2 is additionally characterized by dysmorphic features, hepatosplenomegaly, skeletal, renal, and cardiac involvement, and early onset subtypes—hydrops fetalis. Sialidosis type 2 subtypes include congenital, infantile, and juvenile forms, according to the age of onset and severity of the disease.

Yagi et al. described 3 out of 4 siblings affected with sialidosis type 1, in whom, apart from the LSD, a cancer disease developed. Three kinds of cancer including colon cancer, diffuse large B cell lymphoma, and endometrial cysts, were observed in this sibling at the age of 41, 30, and 33 years of life [75]. This susceptibility of cells with sialidosis type 1 to carcinogenesis has already been described in mouse colon adenocarcinoma cells and human colon cancer tissue, in which an enhanced metastatic ability and diminished expression level of NEU1 mRNA were observed [75,76] cited after [77].

## 7. Carriers of Lysosomal Diseases

Oncologic disease is found not only in patients with a lysosomal storage disorder but also in carriers. Shin et al. found that in heterozygotes for potentially pathogenic variants (PPVs) in LSD genes, there exists a possibility of earlier cancer diagnosis. Especially five cancer-gene pairs were identified: (1) for pancreatic adenocarcinoma PACA-*MAN2B1*, PACA-*GALNS*, PACA-*GUSB* (2) for cutaneous melanoma SKCM–*NPC2*, (3) for chronic myeloid disorder CMDI–*SGSH*, (where *PACA* is an abbreviation for pancreatic adenocarcinoma, *MAN2B1*—the gene for alfa-mannosidosis, *GALNS*—the gene for mucopolysaccharidosis type IV A, *GUSB*—the gene for mucopolysaccharidosis type VII, SKCM—for cutaneous melanoma, *NPC2*—gene for Niemann-Pick disease type C, CMDI—for chronic myeloid disorder, *SGSH*—gene for mucopolysaccharidosis type III A) [78]. Moreover, this study showed that in PPV carriers, when compared with wild-type individuals, not only the risk of cancer is increased, but also cancer risk is higher in individuals with a greater number of PPVs, cancer develops earlier in PPV carriers, and the transcriptional misregulation of cancer-promoting signaling pathways might underlie the oncogenic contribution of PPVs [78].

Shin et al. also performed a population structure–adjusted optimal sequence kernel association test (SKAT-O), whose results are summarized in Table 2. Thirty-seven significantly associated cancer–gene pairs and four genes (*GBA1*, *SGSH*, *HEXA*, and *CLN3*) with a Pan-Cancer association were identified (Pan-Cancer is a cohort of 2567 patients with cancer) [78]. Interestingly, cancer-gene pairs with the highest prevalence ratio included:

(1) *CLN3*-osteosarcoma (prevalence ratio 52.0);
(2) *CLN3*-myeloproliferative neoplasm (32.5);
(3) *GUSB*-pancreas neuroendocrine carcinoma (61.8);
(4) *NEU1*-uterine cervix squamous cell carcinoma (46.4);
(5) *NEU1*-urinary bladder transitional cell carcinoma (36.3);
(6) *SGSH*-myeloproliferative neoplasm (51.1);
(7) *SGSH*-pancreas neuroendocrine carcinoma (30.9);
(8) *SGSH*-osteosarcoma (30.7);
(9) *SGSH*-breast invasive ductal carcinoma (16.5);
(10) *SGSH*-pancreas adenocarcinoma (15.4).

### 7.1. Gene CLN3 and Neuronal Ceroid Lipofuscinosis Type 3 (CLN3)

The *CLN3* gene is coding for a lysosomal transmembrane protein—CLN3, which earlier used to be called Battenin. CLN3 protein plays various roles in the cell, primarily including participation in anterograde and retrograde trafficking between the Golgi network, endosomes, autophagosomes, lysosomes, and plasma membrane, as well as incorporation of sphingolipids into lipid raft microdomains in the plasma membrane [79], involvement in apoptosis and synaptic transmission. Mutations in the *CLN3* gene lead to the unfunctional CLN3 protein. This results in the accumulation of, mainly, subunit c of the mitochondrial ATP synthase F0, which is the pore-forming, membrane-spanning subunit of the mitochondrial ATP synthase complex. Accumulated compounds form the most often “fingerprint” pattern [79].

Severe visual impairment, leading to vision loss and epilepsy, manifesting as generalized tonic–clonic seizures or complex partial seizures, are the most typical clinical symptoms, which may occur as the first signs of the disease in children between 4 and 10 years of life. Other clinical symptoms include the following:

- motor dysfunction due to extrapyramidal, slight pyramidal, and cerebellar disturbances;
- slowly developing mental retardation;
- dementia becomes profound later in the course of the disease;
- disordered speech leading to dysarthria;
- presence of lipofuscin deposits with the typical pattern of ”fingerprints” and vacuolized lymphocytes in electron microscopy (EM) [80,81].

It has been shown that disturbances in delivering sphingolipids to the cellular membranes, and among them especially GalCer, activates the caspase cascade, which leads to the enhancement of apoptotic events. Thus, the CLN3 protein is involved in the regulation of cancer cell growth [78,82].

El-Sitt et al. summarized the properties of the CLN3 protein, which regulates cell growth and apoptosis. In neuronal cells, this protein transports GalCer from the Golgi apparatus to the lipid rafts building plasma membrane through the lysosomes. It has been established that in CLN3-deficient cells, GalCer is arrested in the Golgi apparatus and lysosomes, and it does not reach the lipid rafts. As a consequence, the level of ceramide is increased. This stimulates apoptosis [83].

According to Shematorova et al., CLN3 protein interacts with the alfa-1 subunit of ATP1A1 ATPase, most probably as the chaperone, influencing the spatial structure of ATP1A1 in neurons. Changes in the molecular conformation of the alfa-1 subunit of ATP1A1 define its pumping or signaling action and result in the activation of non-receptor tyrosine kinase SRC, which in turn can be involved in the MAPK (mitosis-activated protein kinases) cascades. As a consequence, the transcription of genes coding for growth factors and mitogens can be enhanced, eventually leading to cell death. Shematorova et al. suggest that the molecular mechanism of CLN3 is caused by the disturbed spatial structure of ATP1A1 subunit alfa-1 due to the incorrect structure of CLN3 protein [84].

### 7.2. Gene GUSB and Mucopolysaccharidosis Type VII (Sly Disease)

The gene *GUSB* encodes a lysosomal hydrolase beta-glucuronidase, which is involved in the degradation of glycosaminoglycans (GAGs), including heparan sulfate (HS), dermatan sulfate (DS), and chondroitin-4,6-sulfate (CS). Mutations in *GUSB* gene lead to mucopolysaccharidosis type VII (MPS VII, Sly disease), a lysosomal storage disease inherited in an autosomal recessive manner.

Similarly to other MPSs, patients affected with MPS VII present with mental retardation, short stature, coarse facial features, spinal abnormalities, dysostosis multiplex, and enlargement of the liver and spleen. In severe forms of this disease, hydrops fetalis may be observed.

In animal models, disturbed autophagy processes were demonstrated. Additionally, increased expression of genes involved in innate immune system and inflammation was observed in brains from the mouse model of MPS VII [85].

### 7.3. Gene SGSH and Mucopolysaccharidosis Type IIIA (Sanfilippo Syndrome Type A)

The *SGSH* gene is coding for a lysosomal hydrolase N-Sulfoglucosamine Sulfohydrolase, also known as sulfamidase or heparan-N-sulfate sulfatase, which catalyzes the cleavage of a bond between glucosaminide and an N-linked sulfate residue of heparan sulfate (HS). Lack of sulfamidase activity results in mucopolysaccharidosis type IIIA (MPS IIIA), which is also named Sanfilippo syndrome type A.

This rare lysosomal storage disease is a neurodegenerative disorder accompanied by mild somatic symptoms: coarse face, thick stiff hair, hirsutism, hepatosplenomegaly, dysostosis multiplex, thickened bones of calvaria, and joint stiffness. Neurologic features include mental retardation, hyperactivity, seizures, sleep, and behavioral problems. The onset of clinical symptoms is between 2 and 6 years.

As a consequence of the deficient activity of sulfamidase, the heparan sulfate is stored and other compounds due to secondary storage. Experimental data indicate that accumulated undigested materials stimulate oxidative stress and disorganize processes of autophagy [85]. Shin et al. suggest that since oxidative stress is a key mediator of cancer cell growth, invasiveness, and angiogenesis, carriers of pathogenic *SGSH* variants may be exposed to an elevated cancer risk because of persistent cellular exposure to oxidative stress [78].

## 8. Conclusions

- Most often, cancer is found in individuals affected with Gaucher, Fabry, Niemann-Pick type A and B diseases, alfa-mannosidosis, and sialidosis.
- Carriers of pathogenic variants in LSD genes and patients affected with some lysosomal diseases are at an increased risk for cancer, especially carriers of variants in *CLN3*, *SGSH*, *GUSB*, *NEU1* genes;
- There are controversial results from studies on the frequency of oncological diseases in LSD patients.

## Figures and Tables

**Table 1:** Lysosomal storage diseases are described in this article (on the basis of [33], modified and actualized).

Disease	Deficient Enzyme or Protein	Inheritance	Gene
**Sphingolipidoses**			
Fabry disease	α-Galactosidase A	AR	*GLA*
Gaucher disease -types I, II and III	β-Glucosidase	AR	*GBA1*
Gaucher disease, atypical	Saposin C defect	AR	*PSAP*
Niemann–Pick A and B	Sphingomyelinase	AR	*SMPD1*
**Mucopolysaccharidoses (MPS)**			
MPS IIIA, Sanfilippo A	Heparan N-sulfatase	AR	*SGSH*
MPS VII, Sly	β-Glucuronidase	AR	*GUSB*
**Glycoproteinoses (Oligosaccharidoses)**			
α-Mannosidosis	α-D-Mannosidase	AR	*MAN2B1*
Sialidosis I/II (Mucolipidosis I)	Neuraminidase (Sialidase 1)	AR	*NEU1*
**Neuronal Ceroid Lipofuscinoses (CLNS)**			
CLN3 disease (Juvenile NCL, JNCL Batten disease)	CLN3, lysosomal, and/or Golgi transmembrane protein	AR	*CLN3*

Abbreviations: AR, autosomal recessive.

**Table 2:** Results of population structure–adjusted optimal sequence kernel association test (SKAT-O) performed in carriers for potentially pathogenic variants (PPVs) in genes causing lysosomal storage diseases (from [78], modified).

Gene Symbol	Lysosomal Storage Disease	Associated Histological Cohort	Relative Prevalence Ratio *
*ARSA*	Metachromatic leukodystrophy	Kidney-RCC ^1^	4.8
*CLN3*	Neuronal ceroid lipofuscinosis type 3	Bone-Osteosarc	52.0
		Myeloid-MPN	32.5
		Breast-AdenoCA	11.7
		Ovary-AdenoCA	4.1
		Pan-Cancer	3.1
*GAA*	Pompe disease	Myeloid-MPN	9.9
		Panc-Endocrine	2.6
		CNS-Medullo	1.5
*GALC*	Krabbe disease	Skin-Melanoma	2.3
*GBA1*	Gaucher disease	Pan-Cancer	1.3
*GNPTAB*	Mucolipidosis type II/III; pseudo-Hurler polydystrophy; I-cell disease	ColoRect-AdenoCA	10.1
		Panc-Endocrine	15.5
		Uterus-AdenoCA	14.2
*GNS*	Mucopolysaccharidosis type III D	Kidney-RCC	4.4
*GUSB*	Mucopolysaccharidosis type VII	Panc-Endocrine	61.8
*HEXA*	Gangliosidosis GM2; Tay-Sachs disease; Sandhoff disease	Breast-AdenoCA	6.8
		Pan-Cancer	2.2
*HEXB*		Liver-HCC	3.5
*HGSNAT*	Mucopolysaccharidosis type III C	CNS-Medullo	8.1
		Head-SCC	8.1
		Ovary-AdenoCA	6.2
		Kidney-RCC	4.8
*IDUA*	Mucopolysaccharidosis type I	Bone-Osteosarc	11.9
		Panc-AdenoCA	7.2
*IDS*	Mucopolysaccharidosis type II; Hunter syndrome	Lymph-BNHL	4.7
		Prost-AdenoCA	2.5
*MAN2B1*	Alfa-mannosidosis	Panc-AdenoCA	3.9
*NEU1*	sialidosis	Cervix-SCC	46.4
		Bladder-TCC	36.
*NPC1*	Niemann-Pick disease type C	Eso-AdenoCA	11.5
		Skin-Melanoma	6.5
		CNS-Medullo	5.9
		Lung-SCC	5.9
		Ovary-AdenoCA	3.8
*SGSH*	Mucopolysaccharidosis type III A	Myeloid-MPN	51.1
		Panc-Endocrine	30.9
		Bone-Osteosarc	30.7
		Breast-AdenoCA	16.5
		Panc-AdenoCA	15.4
		Pan-Cancer	6.5

^1^ Bladder-TCC—urinary bladder transitional cell carcinoma, Bone-Osteosarc—osteosarcoma, Breast-AdenoCA—breast invasive ductal carcinoma, Cervix-SCC—uterine cervix squamous cell carcinoma, CNS-Medullo—medulloblastoma, ColoRect-AdenoCA—colon and rectum adenocarcinoma, Eso-AdenoCA—esophagus adenocarcinoma, Head-SCC—head and neck squamous cell carcinoma, Kidney-RCC—renal cell carcinoma—clear cell type, Liver-HCC—hepatocellular carcinoma, Lung-SCC—lung squamous cell carcinoma, Lymph-BNHL—non-Hodgkin B-cell lymphoma, Myeloid-MPN—myeloproliferative neoplasm, Ovary-AdenoCA—ovary adenocarcinoma, Pan-Cancer—a cohort of 2567 patients with cancer, Panc-AdenoCA—pancreas adenocarcinoma, Panc-Endocrine—pancreas neuroendocrine carcinoma, Prost-AdenoCA—prostate adenocarcinoma, Skin-Melanoma—skin malignant melanoma, Uterus-AdenoCA—uterus adenocarcinoma. * Relative prevalence ratio is the ratio of prevalence of potentially pathogenic variants (PPV) in patients with cancer divided by the prevalence of PPV in healthy individuals.

## Data Availability

Online Data Bases: –OMIM. Available online: http://www.omim.org/entry/248500 (accessed on 1 August 2024).

## Supplementary Materials

The following supporting information can be downloaded at https://www.mdpi.com/article/10.3390/cells13191664/s1. Supplementary Material File S1 with embedded Tables S1: Lysosomal storage diseases (on the basis of Winchester [2012], modified and actualized); Table S2: The roles of lysosomal peptidases in cancer (on the basis of Kos et al., 2022; below Cat means cathepsin); and Table S3: Examples of cellular pathways with changed expression in cancer cells during progression (on the basis of [Machado et al., 2021]).
