# Supplementary material for: Oncological Aspects of Lysosomal Storage Diseases
Source: Cells. 2024 Oct 8;13(19):1664. doi: 10.3390/cells13191664 (PMC11475748; doi:10.3390/cells13191664)
Supplement: Supplementary file 1 [file cells-13-01664-s001.zip › cells-3168602-supplementary.pdf]

Agnieszka Ługowska

“Oncological aspects of lysosomal storage diseases”.

SUPPLEMENTARY MATERIAL File S1.

As mentioned in the Introduction, lysosomes contain hydrolytic enzymes, among which peptidases are distinguished. These enzymes participate in digestion of proteins and peptides no longer usable for cell metabolism. According to Kos et al. lysosomal peptidases are involved in cancer progression, because they are engaged in crucial processes leading to changes in cell morphology, signaling, migration and invasion, and metastasis. On the other hand, lysosomal peptidases can play a role in processes leading to cancer regression (e.g. apoptosis of tumor cells or antitumor immune responses) [Kos et al., 2022]. Lysosomal participation in the immune regulation of cancers has already been described and it is connected with regulation, transport and degradation of cell surface molecules, such as cytotoxic glycoprotein T lymphocyte antigen-4 (CTLA-4) [Tang et al., 2020]. This mechanism could be an inspiration for novel antitumor therapies.

Kos et al. emphasizes that transformation of cells to their malignant forms leads to dysregulated action of lysosomal peptidases involved in signaling pathways (e.g. cytokine/chemokine signaling), modification of growth factors and receptors relevant for oncologically changed cell growth and proliferation. Lysosomal peptidases play role in several processes resulting in degradation of proteins of extracellular matrix (ECM), like collagen fibers or integrins, or biochemical molecules engaged in proteolytic cascades. These processes include tumor invasion, cell migration, and metastasis [Kos et al., 2022; Tang et al., 2020].

Machado et al. pointed out that enhanced lysosomal exocytosis activates several processes which allow cancer cells to become aggressive and intractable:

1) plasma membrane composition is changed and influences signaling pathways, so that they enable survival and migration;

2) lysosomal hydrolases influence the remodeling of extra cellular matrix and activate stroma resident cells;

3) released exosomes spread signaling molecules to neighboring cells;

4) drug resistance can be developed by promoting the efflux of lysosomotropic chemotherapeutics [Machado et al., 2021].

Lysosomal peptidases participate in another essential process, namely tumor progression by tumor angiogenesis. Proliferation of endothelial cells is enhanced by activation of vascular endothelial factor (VEGF), platelet-derived growth factor, interleukins, and transforming growth factor-beta (TGF-beta). Proteolysis catalyzed by peptidases permits migration and invasion of endothelial cells, results in activation of cytokines, growth factors, as well as pro- and antiangiogenic factors [Kos et al., 2022]. The roles of lysosomal peptidases in cancer are summarized in Supplementary Table S2.

Moreover, the autophagic and degradative activity of lysosomes in cancer cells provides them with nutrients and energy needed for development and progression [45]. It has also been shown that aerobic glycolysis occurring in cancer cells leads to an increase in the intracellular H^+ ions concentration, which can be stabilized by proton pumps present in the lysosomal membrane [Tang et al., 2020].

Tang noticed that in numerous studies increased activity of lysosomal enzymes was observed in cancer cells as well as activation of kinesin and dynein, which interfere with lysosomal movement in the cytoplasm. This process was connected with cancer cell metastasis and drug resistance [Tang et al., 2020]. In the review article on the impact of lysosome in cancer cells, Tang et al. recognize more aspects like cancer cells energy metabolism and cancer cell proliferation [Tang et al., 2020]. Machado et al. explain that movement of lysosomes to the plasma membrane of cancer cells is the effect of downregulated expression of the RAB7A and RAB7B genes, which products (Rab7 proteins) play a key role in the regulation of vesicular transport [Machado et al., 2021]. Cellular pathways with changed expression in various types of cancer cells and their connection to lysosomal system are summarized in Supplementary Table S3.

References:

Kos, J.; Mitrović, A.; Perišić Nanut, M.; Pišlar, A. Lysosomal peptidases-intriguing roles in cancer progression and neurodegeneration. FEBS Open Bio. 2022, 12, 708-738. doi: 10.1002/2211-5463.13372.

Tang, T.; Yang, Z.Y.; Wang, D.; Yang, X.Y.; Wang, J.; Li, L.; Wen, Q.; Gao, L.; Bian, X.W.; Yu, S.C. The role of lysosomes in cancer development and progression. Cell Biosci. 2020, 10, 131. doi: 10.1186/s13578-020-00489-x.

Machado, E.R.; Annunziata, I.; van de Vlekkert, D.; Grosveld, G.C.; d'Azzo, A. Lysosomes and Cancer Progression: A Malignant Liaison. Front. Cell Dev. Biol. 2021, 9, 642494. doi: 10.3389/fcell.2021.642494

Agnieszka Ługowska

"Oncological aspects of lysosomal storage diseases".

Supplementary Table S1. Lysosomal storage diseases (on the basis of Winchester [2012], modified and actualized).

Disease	Deficient enzyme or protein	Inheritance	Gene
**Sphingolipidoses**			
GM1-gangliosidosis	β -Galactosidase	AR	*GLB1*
GM2-gangliosidosis<br>Tay–Sachs<br>Sandhoff<br>GM2 activator defect	 β -Hexosaminidase A (α polypeptide)<br>β-Hexosaminidase A and B (β polypeptide)<br>GM2 ganglioside activator	 AR<br>AR<br>AR	 *HEXA*<br>*HEXB*<br>*GM2A*
Fabry disease	α -Galactosidase A	AR	*GLA*
Gaucher disease<br>-types I, II and III<br>Gaucher disease, atypical	 β -Glucosidase<br>Saposin C defect	 AR<br>AR	 *GBA1*<br>*PSAP*
Metachromatic leukodystrophy<br>Metachromatic leukodystrophy	Arylsulfatase A<br>Saposin B	AR<br>AR	*ARSA*<br>*PSAP*
Globoid cell leukodystrophy;<br>Krabbe disease<br>Atypical Krabbe due to saposin A defect	 β -Galactocerebrosidase<br>Saposin A	 AR<br>AR	 *GALC*<br>*PSAP*
Niemann–Pick A and B	Sphingomyelinase	AR	*SMPD1*
Farber	Acid ceramidase	AR	*ASAH1*
Prosaposin deficiency	Prosaposin	AR	*PSAP*
**Mucopolysaccharidoses (MPS)**			
MPS I<br>Hurler (MPS IH)<br>Hurler/Scheie (MPS IH/S)<br>Scheie (MPS IS)	α -L-iduronidase	AR	*IDUA*
MPS II Hunter	Iduronate-2-sulfatase	X-LR	*IDS*
MPS IIIA Sanfilippo A	Heparan N-sulfatase	AR	*SGSH*
MPS IIIB Sanfilippo B	α -N-acetyl-glucosaminidase	AR	*NAGLU*
MPS IIIC Sanfilippo C	Acetyl CoA: α -glucosamine N-acetyl transferase	AR	*HGSNAT*
MPS IIID Sanfilippo D	N-acetylglucosamine-6-sulfatase	AR	*GNS*
MPS IIIE Sanfilippo E	N-glucosamine 3-O-sulfatase (arylsulfatase G or ARSG)*	?	*ARSG*
MPS IVA Morquio A	N-acetylgalactosamine-6-sulfatase	AR	*GALNS*
MPS IVB Morquio B	β -Galactosidase	AR	*GLB1*
MPS VI Maroteaux-Lamy	N-acetylgalactosamine-4-sulfatase (Arylsulfatase B)	AR	*ARSB*
MPS VII Sly	β -Glucuronidase	AR	*GUSB*
MPS IX Natowicz	Hyaluronidase	AR	*HYAL1*
MPS X	Arylsulfatase K	AR	*ARSK*
MPS Plus Syndrome	vacuolar-protein-sorting-associated protein 33A	AR	*VPS33A*
**Glycoproteinoses (Oligosaccharidoses)**			
Aspartylglucosaminuria	Aspartylglucosaminidase	AR	*AGA*
Fucosidosis	α -L-Fucosidase	AR	*FUCA1*
α -Mannosidosis	α -D-Mannosidase	AR	*MAN2B1*
β -Mannosidosis	β -D-Mannosidase	AR	*MANBA*
Sialidosis I/II<br>(Mucolipidosis I)	Neuraminidase (Sialidase 1)	AR	*NEU1*
Schindler/Kanzaki disease	α -N-acetyl-galactosaminidase<br>(α -Galactosidase B)	AR	*NAGA*
Galactosialidosis<br>(PPCA deficiency)	Protective protein / cathepsin A (PPCA)	AR	*CTSA*
**Other enzyme defects**			

Glycogen storage disease type II (Pompe disease)	α -Glucosidase (acid maltase)	AR	*GAA*
Wolman, cholesterol ester storage disease (CESD)	Acid lipase (acid esterase)	AR	*LIPA*
Papillon-Lefèvre	Cathepsin C; Dipeptidyl peptidase I (DPP I)	AR	*CTSC*
Pycnodysostosis	Cathepsin K	AR	*CTSK*
**Defects in post-translational processing of lysosomal enzymes**			
Multiple sulfatase deficiency, mucosulfatidosis	Formylglycine generating enzyme (FGE)	AR	*SUMF1*
Mucolipidosis II α / β<br>(ML II or I-cell)<br>Mucolipidosis III α / β<br>(ML IIIA or pseudo-Hurler polydystrophy)	N-acetylglucosamine-1 phosphotransferase α / β subunit	AR	*GNPTAB*
Mucolipidosis III γ<br>(ML III variant)	N-acetylglucosamine-1 phosphotransferase γ subunit	AR	*GNPTG*
Stuttering (STUT2)	N-acetylglucosamine-1 phosphotransferase α / β and γ subunits<br>N-acetylglucosamine-1-phosphodiester α-N-acetylglucosaminidase (uncovering enzyme)		*GNPTAB*[$]<br>*GNPTG*[$]<br><br>*NAGPA*[$]
**Lysosomal membrane and transport defects**			
Cystinosis	Cystinosin (cystine transporter)	AR	*CTNS*
Sialic acid storage disease Infantile (ISSD)<br>Salla, adult form	Sialin (sialic acid transporter)	AR	*SLC17A5*
Cobalamin F disease<br>(Methylmalonic aciduria and homocystinuria, cblF type)	Cobalamin transporter	AR	*LMBRD1*
Danon	Lysosome-associated membrane protein 2 (LAMP-2)	X-LD	*LAMP2*
LIMP-2 deficiency /Action myoclonus renal failure syndrome	LIMP-2 (Lysosomal integral membrane protein 2 or SCARB2 (Scavenger Receptor Class B)	AR	*SCARB2*
Malignant infantile<br>Osteopetrosis = Osteopetrosis Autosomal Recessive 4	CLCN7, chloride channel 7	AR	*CLCN7*
Osteopetrosis Autosomal Dominant 2	CLCN7, chloride channel 7	AD	*CLCN7*
Osteopetrosis Autosomal Recessive 5 and Infantile Osteopetrosis With Neuroaxonal Dysplasia	OSTM-1, osteopetrosis associated transmembrane protein 1	AR	*OSTM1*
Mucolipidosis IV	Mucolipin-1 (TRPML, transient receptor potential mucolipin)	AR	*MCOLN1*
Niemann–Pick type C1	Niemann–Pick type C1 protein (proton-driven transporter)	AR	*NPC1*
Niemann–Pick type C2	Niemann–Pick type C2 Protein (soluble lysosomal protein)	AR	*NPC2*
**Neuronal ceroid lipofuscinoses (CLNs)**			
CLN1 disease (Infantile NCL, INCL)	Palmitoyl protein thioesterase 1 (PPT1)	AR	*PPT1*
CLN2 disease (Late infantile NCL, LINCL)	Tripeptidyl peptidase 1 (TPP1)	AR	*TPP1*
CLN3 disease (Juvenile NCL, JNCL Batten disease)	CLN3, lysosomal and/or Golgi transmembrane protein	AR	*CLN3*
Recessive Adult NCL<br>(ANCL Kufs)	CLN6 transmembrane protein in ER	AR	*CLN6*

CLN4 disease (Dominant Adult NCL, Parry disease)	CSP α (Cysteine string protein alfa, soluble protein)	AD	*DNAJC5*
CLN5 disease (variant late infantile, vLINCL)	CLN5, soluble lysosomal protein	AR	*CLN5*
CLN6 disease (variant late infantile, vLINCL)	CLN6 transmembrane protein in ER	AR	*CLN6*
CLN7 disease (variant late infantile, vLINCL)	CLN7/MFSD8 (major facilitator superfamily domain-containing protein 8), transporter	AR	*MFSD8*
CLN8 disease (variant late infantile, vLINCL)	CLN8 transmembrane protein in ER	AR	*CLN8*
CLN10 disease (congenital/late infantile)	Cathepsin D	AR	*CTSD*
CLN11 disease	progranulin	AR	*GRN*
CLN12 disease	CLN12 (membrane protein)		*CLN12/ATP13A2*
CLN13 disease (Kufs type B)	Cathepsin F	AR	*CTSF*
CLN14 disease	Potassium Channel Tetramerization Domain-containing Protein 7 (soluble protein)	AR	*CLN14/KCDT7*
**Defects in lysosome and lysosome-related organelle biogenesis**			
Chediak-Higashi syndrome	LYST protein (Lysosomal Trafficking Regulator)	AR	*CHS1/ LYST*
Griscelli syndrome type 1	Myosin 5A	AR	*MYO5A*
Griscelli syndrome type 2	Rab27A (soluble GTPase)	AR	*RAB27A*
Griscelli syndrome type 3	Melanophilin	AR	*MLPH*
Hermansky-Pudlak Syndrome:			
Types 1	HPS1 protein, which is a component of three different protein complexes termed biogenesis of lysosome-related organelles complex (BLOC)-3, BLOC4, and BLOC5.	AR	*HPS1*
Type 2	Adaptor protein-3 (AP3)	AR	*AP3B1*
Type 3	HPS3 protein (HPS3 Biogenesis Of Lysosomal Organelles Complex 2 Subunit 1)	AR	*HPS3*
Type 4	HPS4 protein (HPS4 Biogenesis Of Lysosomal Organelles Complex 3 Subunit 2)	AR	*HPS4*
Type 5	HPS5 protein (HPS5 Biogenesis Of Lysosomal Organelles Complex 2 Subunit 2)	AR	*HPS5*
Type 6	HPS6 protein (HPS6 Biogenesis Of Lysosomal Organelles Complex 2 Subunit 3)	AR	*HPS6*
Type 7	Dysbindin protein = Dystrobrevin Binding Protein 1, BLOC-1 Subunit 8	AR	*DTNBP1*
Type 8	HPS8 protein, which is a component of BLOC1S3 (Biogenesis Of Lysosomal Organelles Complex 1 Subunit 3)	AR	*BLOC1S3*
Type 9	Pallidin, which is BLOC1S6 (Biogenesis Of Lysosomal Organelles Complex 1 Subunit 6) component	AR	*PLDN*
Type 10	Delta-adaptin, which is a subunit of the AP3 adaptor-like complex	AR	*AP3D1*
Type 11	Muted protein, which is part of BLOC1S5 (Biogenesis Of Lysosomal Organelles Complex 1 Subunit 5)	AR	*BLOC1S5*

Abbreviations : AR, autosomal recessive; AD, autosomal dominant; X-LD, X-linked dominant; X-LR, X-linked recessive; $ Genetic association to be confirmed; * ARSG deficiency in humans has yet to be uncovered

References:

- Winchester, B. Classification of Lysosomal Storage Diseases. In Lysosomal Storage Disorders, A Practical Guide, 1st ed.; Mehta, A., Winchester, B., Eds.; Wiley-Blackwell: United Kingdom, 2012; pp. 37-46

Agnieszka Ługowska
"Oncological aspects of lysosomal storage diseases".

Supplementary Table S2. The roles of lysosomal peptidases in cancer (on the basis of Kos et al., 2022; below Cat means cathepsin):

**PROCESS**	**CATHEPSIN**
tumor cell growth	CatB, CatL, CatD, CatG
cell proliferation	CatB, CatL, CatC, Legumain, CatD
invasion	CatB, CatL, CatV, CatS, CatK, CatX, legumain, CatD, CatG
epithelial–mesenchymal transition (EMT)	CatB, CatL, CatV, CatS, CatX, legumain, CatE
angiogenesis	CatB, CatL, CatS, CatK, CatH, legumain, CatD, CatG, CatE
tumor progression	CatB, CatK, CatX
apoptosis	CatB, CatL, CatV, CatS, CatK, CatH, CatS, CatX, legumain, CatD, CatG, CatE
metastasis	CatB, CatL, CatS, CatK, CatC, CatX, legumain, CatD
suppression of antitumor immunity	CatB, CatL, CatK, CatX, legumain
hyperproliferation	CatV
cellular crosstalk	CatK
inflammation	CatK
migration	CatH, CatX, CatG, CatD, legumain
adhesion	CatH, CatX
autophagy	CatC, CatD, CatS
cell death	CatB
bone resorption	CatG
cell aggregation	CatG
growth arrest	CatE

Reference:

- Kos, J.; Mitrović, A.; Perišić Nanut, M.; Pišlar, A. Lysosomal peptidases-intriguing roles in cancer progression and neurodegeneration. *FEBS Open Bio*. **2022**, *12*, 708-738. doi: 10.1002/2211-5463.13372.

Agnieszka Ługowska
“Oncological aspects of lysosomal storage diseases”.

Supplementary Table S3. Examples of cellular pathways with changed expression in cancer cells during progression (on the basis of [Machado et al., 2021]).

Cellular pathway or process	Type of cancer	changes
c-MYC/HDAC2-MiT/TFE transcriptional rheostat	colon adenocarcinoma<br>medulloblastoma<br>rhabdomyosarcoma	- promotion of the progression to an aggressive, higher grade state<br>- high levels of c-MYC and HDAC2 in the nucleus result in inhibition of lysosomal biogenesis and autophagy due to the relocation of TFEB/TFE3 to the cytoplasm
inactivation of mTORC1 and consequent translocation of MiT/TFE to the nucleus	pancreatic ductal adenocarcinoma	- enhanced autophagy and lysosomal catabolism<br>- relevant level of amino acids for cell growth
accumulation and stabilization of MITF	melanoma	- endo-lysosomal biogenesis<br>- increase in biogenesis of late endosomes/multivesicular bodies (MVBs)<br>- enhanced WNT signaling due to sequestration of GSK3
- overexpression of TFEB<br>- WNT/β-catenin signaling pathway	papillary renal carcinoma with liver metastasis	activation of β-catenin induces enhanced expression of target genes, including c-MYC
chromosome translocations involving the MiT/TFE members	- melanoma<br>- clear cell sarcoma of the tendon sheath<br>- perivascular epithelioid cell tumor<br>- alveolar soft part sarcoma of the soft tissue<br>- non-small cell lung cancer<br>- renal cell carcinoma	overexpression of the fused genes

Reference:
Machado, E.R.; Annunziata, I.; van de Vlekkert, D.; Grosveld, G.C.; d’Azzo, A. Lysosomes and Cancer Progression: A Malignant Liaison. *Front. Cell Dev. Biol.* **2021**, *9,* 642494. doi: 10.3389/fcell.2021.642494
